# The Physicochemical and Functional Properties of Biosurfactants: A Review

**DOI:** 10.3390/molecules29112544

**Published:** 2024-05-28

**Authors:** Salome Dini, Alaa El-Din A. Bekhit, Shahin Roohinejad, Jim M. Vale, Dominic Agyei

**Affiliations:** 1 Department of Food Science, University of Otago, Dunedin 9054, New Zealand; dinsa022@student.otago.ac.nz (S.D.); aladin.bekhit@otago.ac.nz (A.E.-D.A.B.); 2 Research and Development Division, Zoom Essence Inc., 1131 Victory Place, Hebron, KY 41048, USAjvale@zoomessence.com (J.M.V.)

**Keywords:** bio-surfactants, lipopeptides, glycolipids, petroleum-based surfactants, synergistic effects, bioactive properties, critical micelle concentration

## Abstract

Surfactants, also known as surface-active agents, have emerged as an important class of compounds with a wide range of applications. However, the use of chemical-derived surfactants must be restricted due to their potential adverse impact on the ecosystem and the health of human and other living organisms. In the past few years, there has been a growing inclination towards natural-derived alternatives, particularly microbial surfactants, as substitutes for synthetic or chemical-based counterparts. Microbial biosurfactants are abundantly found in bacterial species, predominantly *Bacillus* spp. and *Pseudomonas* spp. The chemical structures of biosurfactants involve the complexation of lipids with carbohydrates (glycolipoproteins and glycolipids), peptides (lipopeptides), and phosphates (phospholipids). Lipopeptides, in particular, have been the subject of extensive research due to their versatile properties, including emulsifying, antimicrobial, anticancer, and anti-inflammatory properties. This review provides an update on research progress in the classification of surfactants. Furthermore, it explores various bacterial biosurfactants and their functionalities, along with their advantages over synthetic surfactants. Finally, the potential applications of these biosurfactants in many industries and insights into future research directions are discussed.

## 1. Introduction

Surfactants are amphiphilic molecules that possess both hydrophilic and hydrophobic moieties and are frequently referred to in the literature as surface-active agents. The non-polar part of a surfactant is typically comprised of a hydrocarbon chain, while the polar part (hydrophilic head group) consists of peptides, carbohydrates, phosphates, and proteins [1,2]. This unique molecular structure enables surfactants to possess a wide range of properties, namely their capacity to reduce the surface and interfacial tensions between various phases: liquid–solid, liquid–air, and liquid–liquid. The extent of this reduction is contingent upon the molecular structure of the surfactants [3] and the concentration. The molecular organization of the surfactants, i.e., the micelles, occurs at a specific concentration, which is known as the critical micelle concentration (CMC). This concentration reflects the point at which the surfactant has its lowest stable surface tension. Further increasing the surfactant concentration after this point cannot decrease the surface tension as it has reached its minimum [4]. This parameter can be estimated using various approaches, including high-resolution ultrasound spectroscopy, spectrofluorimetry, conductimetry, and densimetry. Subsequently, the results obtained from these approaches are analyzed using mathematical methods such as a linear regression model for tensiometry or a nonlinear regression model for spectrofluorimetry [5]. Surfactants with the lowest CMC and surface tension are economically favorable for use in industrial processes [6].

**Figure 1:**
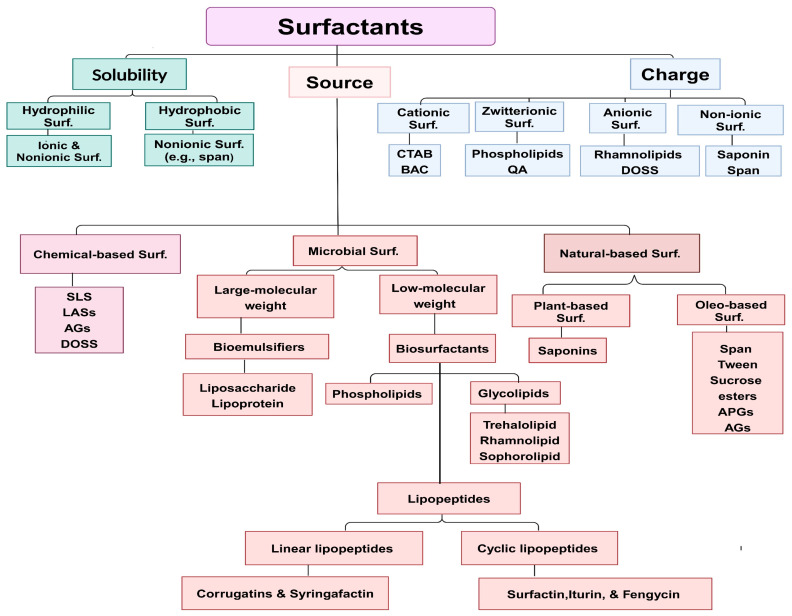
Comprehensive classification of surfactants; cationic surfactants (e.g., hexadecyltrimethylammonium bromide (CTAB), benzalkonium chloride (BAC), and quaternary ammonium (QA)): release positively charged ions when dissolved in water; zwitterionic surfactants (e.g., phospholipids, quaternary ammonium (QA), and lauryldimethylamine oxide (LADO)): containing both functionalities of cationic and anionic surfactants; anionic surfactants (e.g., rhamnolipids, dioctyl sodium sulfosuccinate (DOSS), linear alkylbenzene sulfonates (LASs), sodium lauryl ether sulfate (SLES), and sodium lauryl sulfate (SLS)): release negatively charged ions when dissolved in water; non-ionic surfactants (e.g., saponin, sorbitan esters or span, alkyl polyglycosides (APGs), alkyl glucamide (AGs), sucrose esters, and triphenylmethane (TPM)): non-charged ions in the head; lipophilic surfactants: dissolve in fats; hydrophilic surfactants: dissolve in water. Flowchart created with BioRende.com.

Surfactants exhibit a wide range of potential applications, including antiadhesive and wetting activities, foaming, viscosity reduction, pore-forming capacity, dispersing, emulsification/de-emulsification, solubilizing, and mobilizing properties. These versatile characteristics allow surfactants to be utilized in various industries such as laundry, cosmetics, pharmaceuticals, agriculture, environment, and food. The commercial significance of surfactants is evident from the growing trend of their production and wide range of industrial applications. The estimated global demand for surfactants in 2018 was approximately 17 million metric tons, with a corresponding market value of approximately USD 39 billion [7]. The surfactant sales are expected to increase sharply from USD 41.22 billion in 2021 to USD 57.81 billion in 2028 [8]. Surfactants can be classified according to various criteria (see Figure 1): (1) source (chemical- and natural-based surfactants); (2) headgroup charge (anionic, cationic, non-ionic, and zwitterionic surfactants); and (3) solubility (water and lipid solubility) [9]. It is reasonable to anticipate diverse properties and biological activities among various biosurfactants due to their diverse structures. Therefore, this review primarily focuses on exploring the functional properties of biosurfactants (see Table 1) and their chemical and natural origins and discusses their advantages and disadvantages. Finally, the review presents potential applications of biosurfactants in various industries.

## 2. Surfactant Classifications

### 2.1. Synthetic-Based Surfactants

Synthetic/petroleum-based surfactants are made from non-renewable resources such as crude oil (e.g., paraffinic oil and aromatics) and natural gas (e.g., propylene and ethylene). These surfactants dominate the commercial surfactant market in most industrialized countries, accounting for approximately 90% of the market share due to their high productivity and low cost [36].

The main petroleum-based surfactants include alkyl aryl sulfonate (i.e., linear alkylbenzene sulfonates (LABS), alkylaryl monosulfonate (AMS), and alkylaryl disulfonate (ADS)), α-olefin sulfonates, alkane sulfonate, sulfosuccinate, and quaternary ammonium salts (QAS). These surfactants are achieved through complex processes, including polymerization, alkylation, ethoxylation, and sulfation [37,38]. Most of the surfactants available in commercial products are derived from petroleum, posing significant environmental concern due to the residues they leave behind. These petroleum-based surfactants have lower biodegradability and can easily penetrate cell membranes, posing a serious problem for microorganisms. Therefore, natural-based surfactants, particularly microbial surfactants/biosurfactants, are considered to be a promising alternative to chemical surfactants [39]. Another drawback of petroleum-based surfactants is their contribution to eutrophication, which arises from the presence of phosphates in the structure of these surfactants. Eutrophication leads to oxygen depletion in water due to the massive growth of algae and other plankton, which causes the suffocation of aquatic organisms. This has a negative impact on humans as it results in the loss or extinction of desirable aquatic species. Eutrophication also leads to a decrease in species diversity, increased plant and animal biomass, higher turbidity, accelerated sedimentation, and shortened lake lifespan [40]. Globally, more than 15 million tons of surfactants are utilized annually, with an estimated 60% of these substances ultimately reaching the aquatic environment. Although these compounds are known to be toxic and have severe and long-lasting impacts on both the environment and organisms, they are still in high demand by the manufacturing sector due to their economic effectiveness, high yield, and customizable synthesis [38].

Continuous advancements in research have led scientists to the development of numerous biodegradable petroleum-derived surfactants, including liner alkylbenzene sulfonate (LAS) and paraffin sulfonates. LAS, an anionic petroleum-based surfactant, is presently the most widely used surfactants in industrial applications. It constitutes approximately 25% of the surfactants found in typical household products [41]. Despite its advantages such as affordability, low CMC (around 0.1 mg/L), and good water solubility, LAS still has a notable drawback: relatively low tolerance to hard water. This characteristic is particularly crucial for the laundry industry [42].

### 2.2. Natural-Based Surfactants

Natural-based surfactants can be classified into two categories: those synthesized using renewable raw materials (such as plants, animals, and marine sources) and those produced from the biological processes of microorganisms (biosurfactant). The former is referred to as oleo/bio-based biosurfactants and encompasses fatty alcohol sulfates, fatty acid methyl esters (FAMEs), sucroesters, alkyl polyglycosides (APGs), and fatty acid glucamides, among others. The main materials for producing this type of surfactant are classified into triglycerides (e.g., tallow), carbohydrates (e.g., sorbitol and glucose), and fatty alcohols (e.g., those from palm kernel, rapeseed and coconut oil). To produce fatty alcohols, plant oils need to undergo several chemical processes, including esterification, hydrogenation, and distillation [38].

Various saccharides, such as sucrose, glucose, fructose, mannose, galactose, lactose, xylose, and starch, in both liquid and solid forms, can be used to produce APGs. However, glucose and starch are more commonly utilized due to their widespread availability and affordability. APGs are non-ionic surfactants derived from sugar. They constitute 40% of the world’s surfactant consumption. APG surfactants are characterized by their saccharide units, which impart hydrophilic properties, and alkyl groups derived from fatty acids, which contribute to their hydrophobic nature. This surfactant exhibits variations in alkyl chain length, including linear and mono-branched chains, as well as differences in the degree of saccharide polymerization [43].

Few surfactants, such as saponin—as secondary metabolites—can be produced by certain plant species: *Saponaria officinalis* L., *Allium nigrum* L., and *Panax notoginseng*. These natural products provide a wide range of protective mechanisms in plants against pathogens and insects, as well as enabling them to respond to environmental stresses [44]. Saponins can be divided into two groups depending on the water-soluble sugar groups linked to triterpenoid (C30) or a lipophilic steroid (C27) moiety [45]. Saponins can be separated and precisely identified using electrospray ionization–mass spectra (ESI-MS), nuclear magnetic resonance (NMR, both ^1^H and ^13^C) data [44]. Due to their high surface activity and self-assembly properties, they have become potential carriers for drug delivery systems. For example, the combination of drugs with saponins enhances both their solubility and bioavailability [46]. Saponin surfactants can serve as substitutes for conventional surfactants (e.g., tween 20 and Triton X-100) in microemulsion delivery systems, effectively promoting and stabilizing emulsification [47]. In the food industry, certain saponins can serve as food additives. For example, quillaja saponins (QS) possess the capability to form micelles that encapsulate fat-soluble lutein esters, which are natural food colorants. This formulation enhances solubility and facilitates the incorporation of lutein esters into food matrices [48]. Despite their advantages, the application of saponins still faces certain limitations, such as high cost, applicability, and the potential for hemolysis [46].

Two categories are used to classify biosurfactants based on their molecular mass: low- and high-molecular-mass microbial products. Low-molecular-weight lipopeptides, glycolipids, and phospholipids are commonly considered as biosurfactants, while high-molecular-weight lipoproteins and lipopolysaccharides are considered bioemulsifiers. Larger molecular mass microbial products are known for their emulsifying properties, whereas biosurfactants are recognized for their ability to reduce surface or interfacial tension [49,50]. In addition to molecular weight, they can be distinguished based on their chemical structures. Biosurfactants are composed of hydrophilic sugars, amino acids, fatty acids, and functional groups such as carboxylic acids. On the other hand, bio-emulsifiers consist of complex mixtures of proteins, heteropolysaccharides, lipopolysaccharides, and lipoproteins [51].

Biosurfactant-producing microorganisms can generally be found in marine ecosystems (e.g., algal species) [52], land (e.g., sediment, soil, and sludge), extreme environments (e.g., oil reservoirs), food products (e.g., dairy products, fermented foods, and raw honey) [53,54,55,56], and contaminated environments (e.g., waste water and oil-contaminated soil) [57]. These microorganisms are capable of surviving in a wide range of temperatures, pH levels, and salinity conditions [58].

**Figure 2:**
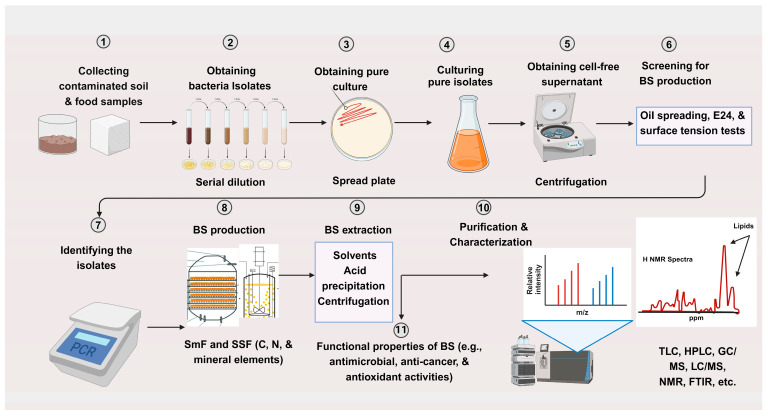
Graphical illustration of biosurfactants produced by bacteria species: bacteria isolation and purification, screening methods, bacteria identification, production, purification, identification, and functional properties. Abbreviations: BS: biosurfactant; E24: Emulsification index test; SmF: submerged fermentation; SSF: solid-state fermentation; C: carbon; N: nitrogen; TLC: thin layer chromatography; HPLC: high-performance liquid chromatography; FTIR: Fourier transform infrared spectroscopy; LC/MS: liquid chromatography–mass spectrometry; GC/MS: gas chromatography/mass spectrometry; NMR: nuclear magnetic resonance. Created with BioRender.com.

The majority of biosurfactants are synthesized extracellularly as lipopeptides and glycolipids, although they could be bound to the plasma membrane, mostly glycolipopeptides [9]. The major biosurfactant producers include *Bacillus* spp., *Pseudomonas* spp., *Arthrobacter* sp., and *Candida* spp. Biosurfactant production involves fermentation, bioreactor systems, and genetic engineering techniques (see Figure 2). The selection of a suitable substrate is crucial as it contributes to approximately 50% of the total production cost. Physicochemical parameters, including carbon (C) and nitrogen (N) sources, C:N ratios, and environmental factors such as the pH and aeration rate, should also be considered [36,59]. To reduce production costs, utilizing low-cost materials like agricultural by-products (e.g., corn steep liquor and orange peel), food waste (e.g., frying oil, molasses, and cheese whey), and dairy waste is a viable option [60,61,62,63].

Culture supernatant secreted from microorganisms to confirm the presence of biosurfactant is commonly detected using various qualitative and semi-quantitative techniques (see Table 2) including emulsion index (E24), drop-collapse, microwell plate, oil spreading, and haemolytic tests, among others [64]. Colorimetric assays, such as orcinol and Bradford protein assays, offer indirect methods for quantifying sugars and proteins, respectively. However, when it comes to quantifying biosurfactants precisely produced by microorganisms, the complex nature of these compounds, which include various congers, isomers, and homologues, poses a significant challenge. High-performance liquid chromatography (HPLC) is considered a feasible and accurate technique for separating individual biosurfactant compounds. This involves first obtaining the cell-free supernatant through centrifugation, followed by purification using a combination of acidification and solvent extraction [65]. The combination of solvent extraction with UPLC-MS is a powerful, accurate, and rapid method for characterizing biosurfactants from bacterial media [66]. *Bacillus* spp. is known for its co-production of diverse families of lipopeptides, encompassing various homologues and isoforms. Previous reports [67,68] have indicated that electrospray ionization (ESI) tandem mass spectrometry has the capability to differentiate between different homologues and isoforms.

Recently, Zhang et al. [69] fully addressed biosurfactant production, the synthesis pathway, and the purification and characterization of biosurfactants. While chemical surfactants (e.g., sodium dodecyl sulfate (SDS) and Triton X-100) are cost-effective and readily available, their environmental impact has motivated scientists to search for more environmentally friendly alternatives [3]. Biosurfactants have demonstrated significant interfacial activity. For instance, biosurfactants (i.e., surfactin, fengycin, iturin, and lichenysin) were found to decrease the surface tension of water from 72 to 27 mN/m [70], whereas sodium dodecyl benzene sulfonate (SDBS), a commonly used synthetic surfactant, reduced the surface tension of water to 33 mN/m [71].

**Figure 3:**
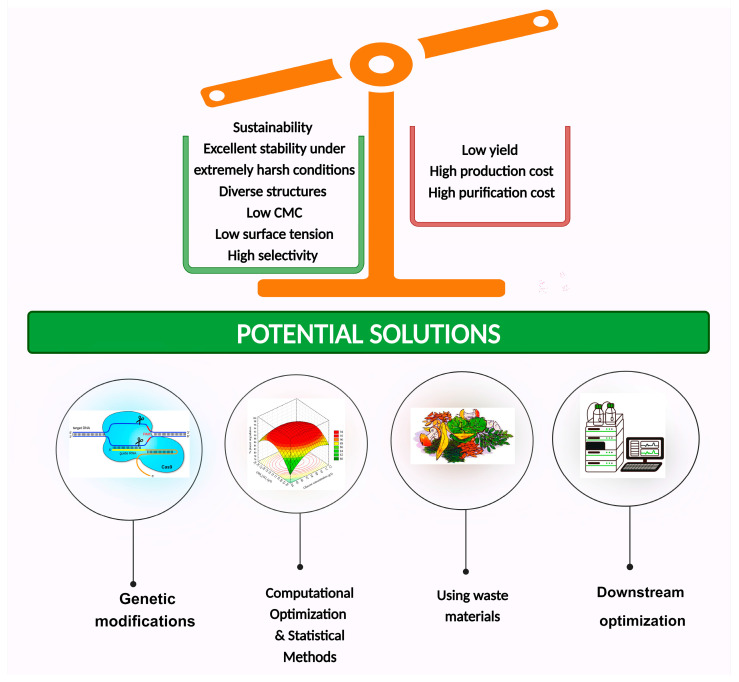
The merits and demerits of microbial surfactants. Created with BioRender.com.

Biosurfactants have several advantages over synthetic counterparts (see Figure 3), including easier biodegradability, greater environmental compatibility, superior foaming capabilities, increased specificity, enhanced efficiency at extreme temperatures, pH levels, and salinity, as well as lower toxicity. For example, the biodegradability of the biosurfactants (e.g., sophorolipids) reached 61% after 8 days of cultivation, whereas the synthetic surfactants (e.g., sodium dodecyl sulfate (SDS)) did not show biodegradability after 8 days [72,73]. More importantly, due to growing concern over environmental issues of petrochemical surfactants, interest has shifted towards the development and promotion of the use of biosurfactants [74,75]. Biosurfactants currently make up approximately 10% of the world’s surfactant production. These versatile organic surfactants find applications in a wide range of industries, including petroleum, food, pharmaceuticals, medicine, and agriculture. Some commercially available products that contain antimicrobial biosurfactants include Sopholiance facial cleanser (Givaudan Active Beauty, Paris, France), Kanebo moisturizer (Kanebo Cosmetics, Tokyo, Japan), Relipidium body moisturizer (BASF, Monheim, Germany) [76,77], and rhamnolipid by Jeneil Biosurfactant Corp [78]. Demand for sustainable surfactants was USD 1.2 billion in 2022 and is forecast to increase to USD 2.3 billion by 2028 https://www.marketsandmarkets.com (accessed on 30 January 2024).

**Figure 4:**
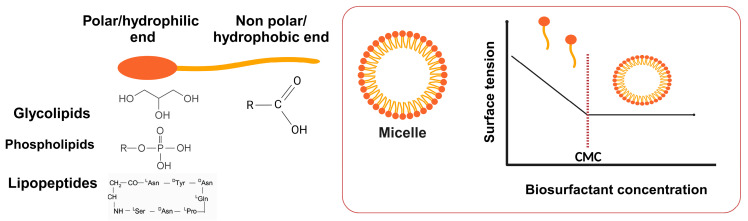
Structural features of microbial surfactants and their micelles. Created with BioRender.com.

There are four major classes of biosurfactants: glycolipids, lipopeptides, phospholipids, and glycolipoprotein (see Figure 4).

#### 2.2.1. Glycolipids

Glycolipids are characterized by carbohydrate (e.g., rhamnose and sophorose units) and fatty acid (e.g., β-hydroxydecanoic acid unit) [79]. They can be broadly divided into three main classes by their polar tails: rhamnolipids, sophorolipids and trehalolipids. However, other glycolipid classes, such as rhamnose lipids, trehalose lipids, sophorose lipids, and diglycosyl diglycerides, are less abundant [80,81]. The most well-known glycolipid-producing microorganisms are *Pseudomonas* spp., *Rhodococcus* spp., *Arthrobacter* spp., *Starmerella bombicola*, and *Candida* spp. [72].

#### 2.2.2. Lipopeptides

Lipopeptides, representing a significant category among microbial surfactants, consist of hydrophilic peptides attached to hydrophobic fatty acids and lipids. *Bacillus* and *Pseudomonas* are valuable since they are known to produce lipopeptides with a wide range of molecular structures and activities. The most abundant lipopeptides produced by *Bacillus* are grouped into surfactin, iturin and fengycin families. They are differentiated based on their amino acid sequence, peptide cyclization and length, and the types of fatty acids. Surfactin consists of C13–C16 hydroxy fatty acid chain and heptapeptides. To date, over 30 variants (e.g., esperin, pumilacidins, and surfactin A–D) have been identified within the surfactin lipopeptide family, encompassing a broad spectrum of different homologues and isomers [82,83]. Iturin families, mainly including iturins, bacillomycins, and mycosubtilin, are formed by the linkage of seven peptides with a β-hydroxy fatty acid chain. Fengycin consists of cyclic decapeptides linked to a β-hydroxy fatty acid chain through an inner ester bond. It contains a number of variants, including fengycin A and B [84]. The most common *Pseudomonas* lipopeptides include viscosin, amphisin, tolaasin, and syringomycin. The viscosin and amphisin families are composed of 9 and 11 amino acids, respectively, linked to 3-hydroxydecanoic acid as their lipid tail. The Syringomycin group consists of nine amino acids, including unusual ones like 2,4-diaminobutyric acid and C-terminal chlorinated threonine residues. In contrast, the tolaasin families exhibit greater diversity due to their composition of 19–25 amino acids and typically 3-hydroxydecanoic acid or 3-hydroxyoctanoic acid as their hydrophobic tail [85]. Recent reviews [69,86,87] fully addressed the production, synthesis pathway, and purification and characterization of lipopeptides. Lipopeptides are considered the most abundant natural biosurfactants [88].

#### 2.2.3. Phospholipids

Several bacteria species synthesize large quantities of phospholipids during growth on n-alkane substrates. The presence of alkane substrates during the growth of hydrocarbon-degrading bacteria (i.e., *Acinetobacter* spp. and *Thiobacillus trioxidanes*) leads to a significant increase in the abundance of phospholipids, which are essential constituents of microbial membranes [77,89]. In one study, the phospholipids produced by *Sphingobacterium* sp. resulted in a reduction in the interfacial tension between water and ethanol at 33 mN/m with a CMC of approximately 0.18 g/L [90]. Limited reports are available regarding the biological activities of phospholipids in comparison to their counterparts.

#### 2.2.4. Glycolipoprotein

The most known biosurfactants found in lactic acid bacteria (e.g., *Lactobacillus acidophilus* and *L. jensenii*) and *Acinetobacter* spp. are glycolipoproteins. The hydrophobic domain includes lipids, and the hydrophilic domain includes carbohydrates and proteins [91]. There are very limited reports available on glycolipoprotein and its functionalities. Jadhav et al. [14] were the first to identify glycolipoprotein in the culture supernatant of *Oceanobacillus* sp. using TLC and FTIR analysis. The authors reported its inhibitory effects against *E. coli* using the disk diffusion method, which is remarkable considering that glycolipoprotein biosurfactants are more effective against Gram-positive bacteria. Consequently, further research is necessary to investigate the correlation between the molecular structure of glycolipoprotein and its antimicrobial activities.

## 3. Physicochemical Properties of Biosurfactants

### 3.1. Emulsifying Activities

Lipopeptides demonstrate emulsion behavior due to ability to stabilize and form emulsions through diverse mechanisms. The emulsifying capacity of lipopeptides can be estimated using emulsification index. In this method, equal concentrations of biosurfactant are added to various hydrocarbon substrates (e.g., olive oil, crude oil, kerosene, and mineral oil) and mixed at maximum speed for a few minutes, followed by storage at room temperature. The resulting emulsions are compared to a negative control (water) and positive controls (Tween-20 or SDS) for differentiation. The emulsifying activity of biosurfactants is usually evaluated over multiple time periods (e.g., 24, 96, and 168 h) to determine the stability of the emulsions. The ratio between the height of an emulsion to the total height of the liquid is used as the emulsion index [92].

According to the literature, several lipopeptides (e.g., surfactin) and phospholipids show emulsifying activities comparable to their commercial counterparts such as sodium dodecyl sulfate (SDS) and Triton X [93,94]. In a study by de Faria, Teodoro-Martinez, de Oliveira Barbosa, Vaz, Silva, Garcia, Tótola, Eberlin, Grossman and Alves [94], surfactin-producing *B. subtilis* LSFM-05 could produce an emulsion that is more stable than SDS and Triton X against kerosene and crude oil after 24 h. However, when compared to other commercial emulsifiers such as xanthan gum (a polysaccharide secreted extracellularly by *Xanthomonas campestris*) and Gum Arabic (extracted from *Acacia* spp.), glycolipoprotein exhibited lower emulsifying activities. For example, the E24 value of the glycolipoprotein against sunflower oil was 76%, whereas Gum Arabic demonstrated a higher value of 98% [95].

These biomolecules play key roles in various industries, particularly in the food industry. Food emulsifiers are capable of dispersing oil in water or vice versa. They contribute to creating the desired texture and structure of food products such as mayonnaise and salad dressings. To choose a food emulsifier, it is crucial to consider factors such as the emulsification time, energy incorporated into the emulsion, and temperature [96], as well as the chemical structure of emulsifiers in terms of the sequence of atoms (such as peptides and proteins) and their configuration (such as α-helix- versus β-sheet-forming peptides). They not only stabilize and form droplets within emulsions but also stabilize bubbles in foams and solids in dispersions [97]. In this context, food emulsifiers aid in the stabilization and formation of emulsions by reducing the surface tension at the oil–water interface [98].

In the micelle form, the hydrophobic part of the biosurfactant molecules is oriented towards the center, creating a core, while the hydrophilic (water-attracting) part faces the surface of the sphere, forming an interface with the surrounding water [99]. In the current food market, natural food emulsifying agents such as lecithin and gum Arabic are widely used. However, their application has encountered certain limitations due to their low resistance to extreme conditions of temperatures and pH in modern food technologies. Based on a study by Lima et al. [100], a lipopeptide from *Candida glabrata* demonstrated the ability to successfully reduce the surface tension from ~50 to 28 mN/m and exhibited the capability to maintain emulsions stable over a broad range of temperatures (0–120 °C), pH values (2–12), and saline concentrations (2–12%). As a result, biosurfactants have emerged as a promising alternative for several applications. Studies have indicated that biosurfactants, such as rhamnolipids, can be employed as emulsifiers to regulate the aggregation of fat globules, stabilize aerated systems, and enhance the texture of fatty products. Specifically, research has explored the utilization of rhamnolipids to enhance the properties of butter, croissants, and frozen pastries [101].

Biosurfactants can be utilized in the bioremediation of oil and metal-contaminated soil. Petroleum hydrocarbon compounds have a propensity to adhere to soil components, making their removal challenging due to their hydrophobic characteristics. Nevertheless, biosurfactants have the ability to emulsify hydrocarbons, resulting in enhanced water solubility, reduced surface tension, and improved displacement of oil substances from soil particles [102]. Bezza and Chirwa [103] reported an 85% oil recovery from contaminated sand using a lipopeptide obtained from *Bacillus subtilis* CN2. The study indicated emulsion indices of around 85% with hexane and cyclohexane. A similar result has been reported by Pandey et al. [104], who observed an enhanced oil recovery using glycolipopeptide. In a study by Giri et al., [29], the emulsification and surface tension reduction activities of a lipopeptide produced by *Bacillus subtilis* VSG4 and *Bacillus licheniformis* VS16 were stable over a wide range of pH values and temperatures (4–10 and 20–100 °C, respectively) for up to 30 min.

### 3.2. Surface Tension Reduction

The formation of micelles, which are assemblies of amphipathic molecules, is an important characteristic of biosurfactants. Micelle formation occurs at a specific concentration of biosurfactants, at which the surface tension reaches its lowest value. As the concentration of the biosurfactant increases, the surface tension decreases, leading to the formation of micelles [4]. In this regard, biosurfactant micelles can be formed in different shapes such as sphere-like, worm-like, and unilamellar bilayers. A spherical shape happens when the solution reaches CMC and may change to a worm-like shape at higher biosurfactant concentrations. Additionally, temperature or other environmental conditions induce the transition from spherical-shaped micelles to micellar vesicles in biosurfactant assemblies. However, these variations can differ under other parameters, e.g., pH, and ionic strength [88]. The effectiveness of biosurfactants and application can be based on the CMC, aggregation number, and surface tension reduction potential [4]. Various techniques are available for determining the CMC and aggregation number of biosurfactants including the THP-based determination method, fluorimetry, surface tension, conductometry, and isothermal titration calorimetry [105,106]. The aggregation number is considered as another important parameter to investigate in a new biosurfactant. The aggregation number refers to the average number of surfactant molecules that form a single micelle, just as CMC has been overreached [88]. In this instance, Liu et al. [107] identified the shape and structure of the surfactin micelles. The micelles demonstrated a core–shell structure, where the core was composed of a hydrocarbon chain and four hydrophobic leucines. Each micelle contained approximately 20 surfactin molecules using small-angle scattering (SAS). The Du Nouy Ring technique is commonly used to qualitatively measure the surface tension value of the biosurfactant using a tensiometer [108].

Clouding behavior can occur when the surfactant is subjected to specific temperatures. This phenomenon leads to phase separation, dividing the surfactant into two distinct phases. The temperature at which phase separation occurs, known as the cloud point (CP), represents the threshold temperature for clouding. As a result, below the CP, the micelle solution remains in a single phase, while above the CP, the solubility of the surfactant in water decreases, leading to the formation of a cloudy dispersion. Clouding can also be induced by changes in pressure or the presence of certain additives. Micelles, which are assemblies of surfactant molecules held together by hydrophobic forces, form at a specific CMC. As the temperature rises, the CMC increases, causing the micelles to collapse. However, the underlying mechanism for the clouding phenomenon in surfactant systems is still not well understood [109,110].

Surfactin is an effective surfactant, reducing water surface tension from 72 mN/m to 27 mN/m at a concentration of 20 µM. Its aggregation activity improves with longer fatty acid chains [111]. Glycolipids are capable of reducing water surface tension from 72 to 28 mN/m [112]. Other studies [113,114] have reported lower surface tensions of approximately 24 mN/m and 26 mN/m for trehalolipid and rhamnolipid, respectively, purified from *Micrococcus luteus* and *P. aeruginosa* PTCC 1340. The CMC for trehalolipid was found to be 0.025 g/L, while for rhamnolipid, it was determined to be 0.09 g/L. It is worth noting that further studies are required to determine the non-pathogenicity of *P. aeruginosa* PTCC 1340. On the contrary, the commonly employed chemical surfactant SDS exhibits a CMC value of 1.8 g/L, leading to a reduction in surface tension from 72.0 mN/m to 37 mN/m [115]. In another study [26], the CMC value of the lipopeptide found in *Halomonas venusta* PHKT was 10~18 times lower than that of citrikleen, SDS, and tetradecyl trimethyl ammonium bromide (TTAB). Ahn et al. [116] observed an inverse relationship between the chain length of fatty acids and surface tension. In their studies, the sophorolipids containing 16 carbons exhibited the lowest surface tension, which was approximately 42 dyne/cm, whereas the surface tension of C10 was measured at 61 dyne/cm.

### 3.3. Biosurfactant Stability

The application of biosurfactants in various fields relies on their stability under different temperature, pH, and salt concentration conditions. Most biosurfactants are considered promising compounds due to their enhanced resistance to adverse conditions encountered in food processing and bioremediation. For instance, in one study, the emulsion formed by glycolipids remained stable at pH 2–10, temperatures of 20–100 °C, and NaCl concentrations of 5–25% *w*/*v* [117]. Similar findings have been found in lipopeptides produced by *Bacillus subtilis* [118]. Moreover, the functional properties of biosurfactants under various conditions should be taken into consideration. In the case of the lipopeptide-derived *Bacillus altitudinis*, it exhibited reduced antifungal activities beyond 100 °C and at both acidic and basic pH levels [119]. Cheffi et al. [26] observed the stability of lipopeptides from *Halomonas venusta* across various salinity (up to 120 g/L), temperature (4–121 °C) and pH (2–12) values by measuring the oil displacement and surface tension.

## 4. Bioactive Properties of Biosurfactants

### 4.1. Antibacterial Activities

The basic mechanisms of antimicrobial action of surfactants include changing membrane permeability and perforation of the cell membrane of the microbial host. Cationic surfactants (e.g., di-N-alkyldimethylammonium halide) have found extensive applications as antimicrobial agents in various fields, including in food and medicine. These effects are attributed to the electrostatic interactions between the positively charged amino acids and the negatively charged bacterial membrane. Additionally, the cationic surfactants interact with lipopolysaccharides (LPSs) and acidic polysaccharide molecules in the case of Gram-negative and -positive bacteria, respectively [120]. In terms of hydrophobicity, the length and number of alkyl chains in the surfactant structure are crucial factors that influence the antimicrobial capacity of the surfactants.

In their study, Pérez et al. [121] modified the composition of the surfactant’s head group and hydrophobicity moieties using renewable materials such as fatty acids and amino acids. The aim was to assess the impact of these modifications on the antibacterial behavior of the surfactants. The results showed that incorporating aromatic amino acids and using the maximum alkyl chain length (C12) led to improved antibacterial activities (more than 50%) against *S. aureus*, *S. epidermidis*, and *B. subtilis*. These improvements were primarily attributed to the disruption of bacterial cell membranes and the subsequent release of cellular material. Furthermore, the attachment of the alkyl chain to the α-amino groups in the modified surfactants resulted in enhanced inhibitory effects against highly resistant bacteria, including Gram-negative bacteria and *L. monocytogenes*.

Likewise, Zhang, et al. [122] demonstrated that the antimicrobial effect of gemini surfactants becomes stronger as the alkyl chain length increases, likely due to the creation of a more hydrophobic environment. The minimum inhibitory concentration (MIC) values of the surfactants against both *E. coli* (MIC = 167.7 μM) and *S. aureus* (2.8 μM) are lower than their corresponding CMC values. At concentrations below the CMC, the surfactant molecules dissolve uniformly in the medium and can easily interact with bacterial cell surfaces through electrostatic and hydrophobic interactions, indicating that the monomeric species of these compounds are responsible for the antimicrobial response. Gemini surfactants have two hydrophobic chains and two hydrophilic heads; thus, they create stronger electrostatic and hydrophobic interactions with the cell membrane compared to dodecyltrimethylammonium bromide (DTAB) and hexadecyltrimethylammonium bromide (CTAB), which have only single-chain surfactant. In gemini surfactants, the two parts are linked together with a spacer, which is commonly a hydrocarbon chain or ring. This linker can be functionalized with a polar group, enhancing the compound’s hydrophilicity. In addition, the hydrophilicity and functionality of the molecules can be increased by the hydrocarbon substituent, which typically contains 8–12 carbons. This structure makes gemini suitable agents in enhanced oil recovery and anti-static clothing, and also as bactericides [123,124].

The results from the study conducted by Zhang et al. [122] indicate that surfactants are more active against Gram-positive bacteria than Gram-negative bacteria. In general, Gram-positive bacteria possess cell membranes primarily consisting of peptidoglycan layers, enabling easy penetration by surfactants. Conversely, Gram-negative bacteria feature external membrane layers rich in lipopolysaccharides and proteins, which act as barriers to biocides. Consequently, Gram-negative bacteria exhibit lower sensitivity compared to their Gram-positive counterparts due to their less permeable outer membranes, a finding supported by prior research cited by Mouafo et al. [125].

The lipoprotein membrane serves a crucial role in maintaining permeability, and any alterations in its selective permeability can result in cellular damage. The hydrocarbon tails of quaternary ammonium surfactants can integrate into bilayers or liposomes, enhancing the permeability of bacterial cell membranes and causing the formation of transient channels or pores [126,127,128]. These channels or pores subsequently heighten membrane permeability, leading to the leakage of cellular contents and disruption of biochemical reactions within the cytoplasm [129].

The antimicrobial actions of the biosurfactants commonly involve the disruption of cell membrane integrity, increased cell membrane permeability, and the inhibition of bacterial protein and ATP synthesis [10,130]. Among microbial surfactants, lipopeptides (e.g., surfactin, iturin, and fengycin) and glycolipids (e.g., rhamnolipids) have demonstrated significant antimicrobial activities. Daptomycin, the first cyclic lipopeptide antibiotic, was approved by the Food and Drug Administration (FDA) in the USA in 2003 as an antibiotic for the treatment of severe blood and skin infections caused by specific Gram-positive microorganisms [131]. Polymyxin is another commercialized lipopeptide antibiotic that is particularly active against Gram-negative bacteria (i.e., *Pseudomonas aeruginosa*). It binds to the LPS in Gram-negative bacteria through electrostatic interactions, utilizing its N-terminal fatty acid tail. This binding leads to bactericidal action by inhibiting the synthesis of the outer membrane [132,133]. However, the usage of polymyxin has been limited due to some toxicological reactions, such as nephrotoxicity. According to previous studies, surfactin has been found to have stronger inhibitory effects against a wide range of bacteria compared to fungal pathogens. Conversely, fengycin and iturin are more dominant in terms of inhibiting fungal growth [134]. Another interesting study [13] observed that the behavior of rhamnolipids against different pathogens differed under different pH environments (from 5 to 9). As a result, the antimicrobial activity of rhamnolipids against *L. monocytogenes* and *S. aureus* (MIC = 9.8–19.5 μg/mL) was found to be pH-dependent, with greater efficacy observed under more acidic conditions (pH 5 and 6). Conversely, the Gram-negative pathogens *Salmonella enterica* and *E. coli* exhibited resistance to rhamnolipids treatment across all pH levels studied. The presence of the outer membrane in Gram-negative bacteria provides selectivity and protection to the cell, which is why they exhibit resistance to anionic surfactants such as rhamnolipids [135]. A study by Ndlovu, Rautenbach, Vosloo, Khan and Khan [66] compared the antibacterial potency of surfactin and rhamnolipids against a broad range of pathogens (e.g., *E. coli*, *Salmonella typhimurium,* and *Enterococcus faecalis*) by determining the diameter of the inhibition zones. The results showed that there were no significant differences in the antibacterial capacity between the two biosurfactants. Furthermore, the antibiofilm activity of rhamnolipids was demonstrated across a range of doses from 0.04 to 1.56 mg/mL, as evidenced by the inhibition of *Bacillus licheniformis* and *Staphylococcus capitis* biofilm formation by up to 90%. This effect was confirmed by a decrease in the biomass of the formed biofilms and a reduction in cell viability [136].

### 4.2. Anti-Fungal Activities

Lipopeptides have demonstrated substantial antifungal activities in several studies by inducing apoptosis at low concentrations, or pore formation in the membrane at high concentrations, affecting the adhesion of pathogens and affecting cell wall integrity [137]. According to previous studies by Kumar and Johri [138], Zhang, et al. [139], fengycin and iturin exhibit the strongest antifungal activities, particularly against phytopathogens (e.g., *Botrytis cinerea, Colletotrichum gloeosporioides,* and *Fusicoccum aromaticum*). These cyclic lipopeptides exhibit antifungal properties by targeting the cell membrane of fungal pathogens, thereby affecting the integrity of the fungal cell wall and modulating membrane permeability. Fengycin binds to lipid layers within biological membranes, inducing structural alterations and increased membrane permeability. Similarly, iturin can modify the target cell membrane in a dose-dependent manner, penetrating the cell wall, damaging the membrane, and disrupting its permeability, ultimately inhibiting fungi through the loss of K^+^ ions. Fengycin and iturin serve as effective antifungal biocontrol agents due to their capacity to enhance cell permeability in fungal hyphae [17].

### 4.3. Anti-Adhesiveness and Anti-Biofilm Activities

The process of bacterial biofilm formation consists of a dynamic sequence that includes an initial attachment stage that progresses to irreversible attachment, followed by biofilm development, biofilm maturation, and biofilm dispersion. The initial attachment of bacterial cells is reversible, accompanied by minimal production of extracellular polymeric substances (EPS). Hence, the first step is the most critical stage to inhibit bacterial proliferation and biofilm formation. Otherwise, the attachment of bacterial cells shifts from reversible to irreversible due to increased EPS production [140].

Biofilms pose a significant challenge in numerous industries including food processing and the medical field. Biofilm-forming foodborne pathogens and spoilage organisms include *Escherichia coli*, *Listeria monocytogenes*, *Salmonella* sp., and *Staphylococcus* spp. [141]. Some conventional antibiofilm agents, such as usnic acid, polymyxin, colistin, and imipenem, may induce allergic reactions or exhibit toxicity. In such scenarios, microbial surfactants serve as a viable and effective alternative [142]. According to a study by de Araujo, et al. [143], the application of rhamnolipids and surfactin reduced the adherence of microbial cells of *L. monocytogenes* to polystyrene and stainless steel. A dose-dependent biofilm formation inhibition activity of bacillomycin D at 25 μg/mL against *C. albicans* has been reported by Tabbene et al. [144]. In another study conducted by Englerova, et al. [145], the administration of 15 mg/mL lipopeptide for 24 h caused the complete inhibition of *Staphylococcus aureus* biofilm. According to Jemil et al. [27], the presence of negatively charged lipopeptides and the negative charge typically found on bacterial surfaces can lead to electrostatic repulsion. This repulsion can occur between polystyrene surfaces coated with lipopeptide biosurfactants and the bacterial membrane, potentially inhibiting the formation of biofilms. Apart from the concentration, treatment temperature, contact duration, and antibiofilm activity can also be influenced by the characteristics the microorganisms. Carvalho, et al. [146] compared the antibiofilm effects of the rhamnolipids under different conditions in terms of temperature (4–25 °C), concentration (2, 5, and 10% (*w*/*v*)), and time of contact (5 and 10 min). The results revealed that contact time was less important than two other tested factors, and the maximum biofilm eradication (*Salmonella enteritidis*, *Escherichia coli,* and *Campylobacter jejuni*) was yielded by the highest temperature and concentration.

### 4.4. Antiviral Activity

Surfactins and fengycins have shown promise as antiviral agents against severe acute respiratory syndrome coronavirus 2 (SARS-CoV-2) [147], pseudorabies virus, Newcastle disease virus, porcine parvovirus, [148], and herpes- and retro-viruses [149]. Most of these viruses are members of the ‘enveloped viruses’, meaning that RNA from intact virus is protected by an envelope. The antiviral efficacy of surfactins is influenced by the number of carbons, meaning that the hydrophobicity of fatty acids increases the inactivation of viruses. Furthermore, the membrane-active surfactant property of surfactin was found to be able to interact with the lipid in the virus envelope, leading to penetration of the lipid bilayer during virus inactivation and the collapse of the viral envelope [150,151]. Lipopeptides from *B. amyloliquefaciens* PPL have been shown to be effective against cucumber mosaic virus [19].

### 4.5. Antioxidant Activities

Synthetic antioxidants, such as butylated hydroxytoluene (BHT), tert-butylhydroquinone (TBHQ), and butylated hydroxyanisole (BHA), are extensively utilized as preservatives in the pharmaceutical and food industries in some parts of the world. Nevertheless, the utilization of these substances is accompanied by adverse effects, raising concerns for public health. As a result, the exploration of biosurfactants as natural antioxidants holds significance in the context of food and pharmaceutical applications [152,153].

According to Jemil, Ben Ayed, Manresa, Nasri and Hmidet [27], lipopeptides found in *Bacillus methylotrophicus* DCS1 demonstrated an approximate 81% DPPH (2,2-diphenyl-1-picrylhydrazyl) radical scavenging activity when tested at a concentration of 1 mg/mL. Surfactin (1 mg/mL) synthesized by *Bacillus subtilis* #309 revealed strong antioxidant activity, determined by DPPH, 2,2-azinobis (3-ethyl-benzothiazoline-6-sulfonic acid (ABTS), and ferric reducing antioxidant power (FRAP). The antioxidant efficacy of surfactin depends on the structure and number of hydroxyl groups on the compound. Surfactin is made up of seven amino acids (L-Glu-L-Leu-D-Leu-L-Val-L-Asp-D-Leu-L-Leu), and the presence of Glu and Asp determines the number of hydroxyl groups, which remains constant across all homologues [28]. Moreover, this property can be influenced by breaking the lactone ring through the hydrolysis and methylation of the peptide loop chain [154].

Another hypothesis suggested by Tabbene, et al. [155] is that the presence of hydrophobic amino acids (Val and Leu) and acidic amino acids (Asp and Glu) in the molecular structure of surfactin contributed to its reducing power. In yet another study, Zouari, et al. [156] observed the potent antioxidant activity of lipopeptides in a dose-dependent manner evidenced by a threefold increase in free radical scavenging capacity of lipopeptides as the concentration was increased from 0.05 mg/mL to 1 mg/mL. Moreover, the antioxidant activities of lipopeptides extracted from *Bacillus* spp. in food have been studied by Jemil, et al. [157] and Kiran, et al. [158]. To promote the utilization of biosurfactants in the food system, further research is needed to assess their safety and stability under severe conditions such as the high temperatures encountered during thermal food processing.

### 4.6. Antiparasitic Activities

Today, it is very important to use environmentally friendly compounds to replace chemical compounds in controlling disease-causing parasites. Previous studies have revealed the antiparasitic activities of surfactin against *Eimeria tenella* [159] and *Nosema ceranae* [160]. A novel lipopeptide, hoshinoamide C, isolated from the cyanobacterium *Caldora. penicillata* inhibited the growth of parasites responsible for malaria and African sleeping sickness [161]. In another study by Soussi et al., surfactin and bacillomycin D from *B. amyloliquefaciens* B84, which is a cause of zoonotic diseases, exhibited antileishmanial activity [162].

### 4.7. Anti-Cancer and Anti-Inflammatory Activities

*Bacillus*-derived lipopeptides (fengycin) have exhibited anticancer activity when tested on different human cancer cell lines, including liver [163] and lung cancer [164]. Cao et al. [165] tested the antitumor properties of surfactin form Bacillus natto TK-1 in MCF-7 cell lines. This lipopeptide was able to generate ROSs, which activates the mediator of JNK and ERK1/2, leading to the induction of apoptosis in the cancer cells. In a HT29 colon cancer cell line, fengycin isolated from *B. subtilis* fmbJ was found to induce apoptosis by affecting the caspase–Bcl-2/Bax pathway, increasing intracellular ROS generation and inducing cell cycle arrest at the G1 phase [166].

In another study, iturin A-like lipopeptides from *B. subtilis* were reported to inhibit colon cancer cells. This effect is linked to the induction of apoptosis via a change the expression of both anti-apoptotic and apoptotic genes (e.g., *bcl-2*, *bax*, and *bad*), along with increasing ROS and Ca^2+^ levels [167]. In addition, the anti-inflammatory effect of surfactin has been confirmed in an in vitro model. This activity is linked to the inhibition of excessive production of pro-inflammatory mediators (e.g., tumor necrosis factor α (TNF-α), interleukin-1 β (IL-1β), and interleukin 6 (IL-6)), ROSs, nitric oxide, and prostaglandin E2 (PGE2) [33,35]. However, there is still a substantial lack of molecular studies directed to better understanding the mechanism of lipopeptides, for their development and application as anti-inflammatory and anti-cancer agents in preclinical and clinical studies.

## 5. Synergetic Effects of Biosurfactants

A binary/mixed surfactant system refers to the combination of different types of surfactants (e.g., biosurfactants and chemically synthesized surfactants) or other active compounds (e.g., chitosan and phenolic compounds), which is preferred due to the provision of less toxic compounds for the environment while also increasing their effectiveness. Several researchers have reported that the combination of biosurfactants and synthetic surfactants leads to higher interfacial activities and significant enhancements in physicochemical properties. Gang et al. [168] reported a significant reduction in interfacial tension (between aqueous solution and crude oil) when using a binary mixture of surfactin and 4-(1-hexyldecyl) benzene sodium sulfonate. Similar findings were observed in a previous study by Shah et al. [169] in which choline laurate (ionic liquid surfactant) and lactonic sophorolipid were mixed. This area of research is particularly interesting for bioremediation purposes, as the use of many chemical dispersants still raises concerns about negative environmental impacts and toxicity.

Previous studies have demonstrated significant anticancer effects of biosurfactants when used in combination with chemotherapy agents. For instance, the combination of iturin A and docetaxel exhibited enhanced anti-cancer efficacy against MDA-MB-231 and MDA-MB-468 breast cancer cells compared to their individual use. This combination approach effectively mitigated docetaxel resistance, a prominent issue in various cancers, particularly breast cancer. Iturin A demonstrated the ability to inhibit the Akt pathway, presenting a novel therapeutic strategy to overcome chemoresistance [170]. The Akt signaling pathway plays a crucial role in the pathogenesis of numerous cancers, and aberrant activation of this pathway results in the dysregulated expression of downstream proteins, ultimately leading to uncontrolled proliferation of cancer cells [171]. A very recent study [172] formulated a new nano emulsion with sophorolipids and eugenol, without any co-surfactant, which exhibited synergistic inhibitory activities against *E. coli* (MIC = 0.5 mg/mL) and *B. cereus* (MIC = 0.125 mg/mL). Their synergetic mechanism involved enhanced cell permeability, alterations in morphology, ATP leakage, and reduced Na^+^-K^+^-ATPase activity. Likewise, the combination of rhamnolipid- and fungal chitosan-derived *Cunninghamella echinulate* exhibited increased antifungal activity (almost two-fold) against *Xanthomonas campestris* compared to the individual administration of chitosan or rhamnolipid [173].

Bao et al. [174] focused on increasing the removal of oil from oily sludge in the petroleum industry using a mixed bio-surfactant (rhamnolipid and sophorolipid) in a thermal washing method. They found that this combination resulted in a considerable reduction in CMC concentration along with the maximum oil removal when using a rhamnolipid/ sophorolipid ratio of 4:6. Similarly, another study [175] reported a synergistic effect when surfactin was combined with SDS. The findings showed that the addition of a small amount of surfactin (0.1 mol%) to an aqueous solution of SDS effectively reduced the CMC of SDS from 10^−3^ to 10^−6^ M without compromising their surface tension activities. This approach proved to be a practical technique for reducing the usage of chemical surfactants.

## 6. Potential Application of Biosurfactant

The potential application of biosurfactants in diverse industries is attributed to their biological activities, as mentioned in Section 4. These functionalities can be influenced by various factors, including their molecular structures. For instance, fengycin—a cyclic lipopeptide—exhibits more potent antifungal properties compared to its counterparts, such as surfactin. According to a study conducted by Sur and Grossfield [176], molecular dynamics simulations revealed that fengycin possesses distinct structural characteristics that enable it to aggregate and selectively bind to fungal membranes, which are abundant in phosphatidylcholine. However, further research is needed to explore the relationship between the structure of biosurfactants and their bioactivities.

Biosurfactant molecules have found widespread applications, particularly in the food industry, as food emulsifiers. Emulsion systems play a vital role in solubilizing and dispersing food formulations, as well as ensuring the stability, appearance, and texture of food products. In this context, food emulsifiers aid in the stabilization and formation of emulsions by reducing the surface tension at the oil–water interface [177,178]. The recent food applications of biosurfactants have been summarized in Table 3. Biosurfactants have aroused considerable interest due to the possibility of acquiring useful products that are tolerant to processing techniques used in industries. Lipopeptides have significant applications in the food industry and can be employed as an antiadhesive foaming agent, wetting agent, solubilizer, and emulsifier [179]. Antimicrobial lipopeptides with high surface activities like surfactin and fengycin possess great potential for use as food preservatives.

Lipopeptides, known as promising microbial surfactants for recovering oil in harsh environmental conditions, have been gaining increasing attention [180]. Several previous scientific reports confirmed the efficacy of lipopeptides when synthesized by *Bacillus* spp. and *Pseudomonas* spp. in bioremediation technology and oil recovery [181,182]. This approach is cost-effective, sustainable, eco-friendly, and non-invasive [183]. Biosurfactants can improve oil recovery by reducing both the surface and interfacial tension between the water and oil interfaces. They can also help maintain the wettability index of a system by regulating the changes in the water–oil interaction [184]. Soils and water contaminated with organic substances and metals are a major environmental issue. Low aqueous solubility of these hydrophobic pollutants in the contaminated sites increases their absorption to soil particles and subsequently prevents the hydrocarbon pollutants from biodegrading micro-organisms. To overcome this issue, the solubility of these contaminants in soil and water can be improved using synthetic or biosurfactants resulting in the maximization of the biodegradation rate and decontamination process [185]. In this case, lipopeptide biosurfactants can enhance the solubility and bioavailability of hydrophobic organic compounds by reducing the interfacial tension and surface. For instance, lipopeptides produced by *Bacillus subtilis* SPB1 enhanced the water solubility of diesel, which was comparable with the tested synthetic surfactants (tween 80 and sodium dodecyl sulfate) [186]. Similar results have been obtained from surfactin and fengycin isolated from *Bacillus mojavensis* A21 [187]. Surfactin and fengycin improved soil washing and is considered an efficient and cost-effective technology for the remediation of soil contaminated by heavy metals such as Pb, Ni, and Zn [188]. Some lipopeptides such as lichenysin [189] and sophorolipid [190] are promising potential candidates in oil recovery because of their high stability at a different temperature, pH, and salinity ranges. Moreover, rhamnolipids and sophorolipids have been found to be successful in hydrocarbon degradation in contaminated soils [73].

**Table 3:** The potential application of biosurfactants in food products.

Biosurfactant-Producing Microorganisms	Biosurfactant Types	Types of Food	Functionalities	Ref.
*Saccharomyces cerevisiae* URM 6670	Glycolipids	Cookie	The biosurfactant replaces the egg yolk, which helps to decrease the concentration of animal fats. The glycolipid showed considerable temperature tolerance from 40 to 400 °C. No considerable changes in the physicochemical parameters of cookies, such as firmness, cohesiveness, and protein and lipid contents	[30]
*Acinetobacter calcoaceticus* RAG-1	NR	Bread	Used to postpone bread staling by preserving its sensory attributes such as hardness and crumb firmness (at a concentration of 0.5%)	[191]
*Bacillus cereus* UCP 1615	NR	Cookie	Used to extend the shelf life of the cookie up to 45 days due to a decrease in moisture content without negatively affecting the hardness, cohesiveness, and sponginess of the product	[192]
*Candida utilis* UFPEDA1009	NR	Cookie	Replacing the isolated biosurfactant with egg yolk in cookie recipes can help reduce the use of animal fat while still maintaining the desired textural elements such as cohesiveness, firmness, and adhesiveness	[193]
*Candida bombicola* URM 3718	Glycolipidic	Cupcake (emulsifier)	A suitable replacement for vegetable oils (with high saturated fatty acids) as an emulsifier without any undesirable effects on the physicochemical parameters of the products such as lipid content and energy levels	[194]
*Candida guilliermondii* (UCP0992)	NR	Mayonnaise As emulsifier	The combination of the biosurfactant (0.5%) and guar gum exhibited significant consistency and texture with no presence of pathogenic microorganisms	[195]
*Saccharomyces cerevisiae* URM 6670	NR	Salad dressing	Used to improve the viscosity properties of the product and remain stable at 4 and 28 °C	[196]
*Bacillus velezensis* BVQ121	Iturin, surfactin, and fengycin	Food packaging	Used to extend the shelf life of the pigeon eggs up to 10 days through inhibition of the pathogen growth of *E. coli*, *E. ictalurid*, and *Salmonella typhimurium*	[197]
*Bacillus licheniformis* MS48	Lipopeptides	Cookies	Used to reduce the gluten content in the cookies while improving their textural and sensory properties	[198]
*Saccharomyces cerevisiae* URM6670	Glycolipids	Muffins	Used to reduce the vegetable oil content with no negative changes in the sensorial parameters of the muffins such as aroma and color	[199]
*Candida utilis*	NR	Salad dressing	Used to enhance the consistency and stability of the salad dressing by adding 7% biosurfactant as an emulsifier	[200]
*Bacillus cereus* UCP 1615	NR	Cookies	Used to keep the textural profiles and energy values and extend the shelf life of the cookies up to 45 days	[192]
*Lactococcus lactis* LNH70	Xylolipids	Fruit juice	Used to preserve the juice for 5 days by reducing bacterial growth	[201]
*Bacillus licheniformis* MS48	Lipopeptides	Yoghurt	Used to enhance the flavor and textural and sensorial characteristics of the final product, as well as to improve the growth and properties of the probiotics (such as *Lactobacillus delbrueckii* subsp. bulgaricus and *Streptococcus thermophilus*)	[202]

NR: not reported.

Pathogenic bacteria and fungi can influence all types of plants, which causes several kinds of diseases, leading to considerable crop yield losses and affecting food safety [203]. A significant number of antimicrobial agents still being used in the agricultural sector are highly toxic and non-biodegradable, harming the environment. Furthermore, many phytopathogens have started to resist these available antimicrobial compounds. Therefore, additional effort is needed to discover safe and effective alternatives to control plant pathogens [134]. Lipopeptides (e.g., iturin A and fengycins) have been described as suitable candidates, as iturin A and fengycins (A and B) could effectively control a variety of plant diseases, such as crown gall caused by *Agrobacterium tumefaciens* [204], *Xanthomonas axonopodis* pv. Vesicatoria infection of tomato and pepper [205], and apple ring rot caused by *Botryosphaeria dothidea* [206]. Iturin A purified from *Bacillus subtilis* ET-1 has been demonstrated to enhance the shelf life of postharvest fruits such as citrus fruits [207] by controlling postharvest pathogens. *Pseudomonas* lipopeptides, particularly orfamide A and xantholysines A and B produced by *Pseudomonas* spp., possess excellent insecticidal activity against *Myzus persicae* and *D. melanogaster* [208]. Additionally, lipopeptide-producing *Bacillus* spp. can remarkably promote seed germination and stimulate plant growth due to increased membrane permeability of seed coat to water, which indirectly accelerates the metabolic processes inside seeds when using the lipopeptide [209]. In addition, several recent studies have demonstrated that rhamnolipids have great potential in agriculture as stimulants of plant immune responses [210], anti-phytopathogenic agents [211], and insecticides [212].

Additionally, antimicrobial lipopeptides are now particularly interesting to scientists since some microorganisms represent resistance to conventional antibiotics. More than 25,000 individuals in Europe and Asia die of bacterial infections annually, and millions of infected people worldwide. So, antibiotic-resistant bacterial infections are an emerging threat to the global public [213]. During the past few years, several classes of lipopeptide antibiotics, namely polymyxin B and daptomycin, have received the FDA’s full approval for treating infections caused by multidrug-resistant pathogens [87,214]. Lipopeptides can also be used in the field of medicine as anti-viral [215], anti-tumor [216], and anti-diabetes [217] agents, as well as lipopeptide-based vaccines [218]. The possibility of foaming by biosurfactant lipopeptides means that lipopeptides would be ideal for incorporation into toothpaste formulation in order to eradicate dental caries and tooth stains and aid in tooth cleaning [219]. Sophorolipids are another type of biosurfactant that has been recommended in toothpaste formulation due to their considerable antibiofilm activities [220]. Lipopeptides exhibit immense promise for integration into various cosmetic formulations thanks to their remarkable surface attributes such as detergency, emulsification, solubilization, dispersion, and foaming capabilities. Moreover, they demonstrate noteworthy anti-wrinkle and moisturizing effects on the skin [221,222]. In comparison to synthetic surfactants, lipopeptides offer several advantages, including reduced irritancy or even anti-irritating effects, superior moisturization, and compatibility with the skin [9]. In particular, surfactin is widely used in anti-wrinkle and cleaning products with excellent washability without skin irritation [223,224]. A study by Ayed et al. showed that lipopeptides synthesized by *Bacillus mojavensis* A21 exhibited considerable antioxidant activity along with significant wound-healing activity in an in vivo system [225]. Moreover, due to the amphiphilic structure of these molecules, they can interact with biological membranes, thereby increasing drug permeability across skin or mucosa [226].

## 7. Conclusions and Future Perspectives

One of the greatest global challenges is to completely replace synthetic compounds with high demands in various industries (environmental, food, and medical industries), such as surfactants, and to seek more sustainable alternatives. In this case, biosurfactants are promising substitutes due to their potential biodegradability and harmlessness [220]. From an economist’s point, both the low yield and high cost of biosurfactant production are major obstacles that hinder their entry into the market at a large scale. To overcome this obstacle, the use of agricultural produce and food waste as a raw material (e.g., carbon and nitrogen resources) to produce value-added products has opened new horizons for contributing to environmental sustainability along with reducing the total cost of production. Biotechnological applications, such as clustered regularly interspaced short palindromic repeat (CRISPR) technology, knockout techniques, and the design of synthetic promoters, can be valuable in enhancing the yield of biosurfactants. Another useful tool for selecting the most suitable bacterial isolates in terms of biosurfactant synthesis is Anti-SMASH. However, previous studies have predominantly focused on the preliminary methods mentioned earlier, such as oil spreading tests and E24, which may not provide accurate results.

Several studies have reported the production of different types of biosurfactants, mainly rhamnolipids [227,228], but few studies have been conducted on producing lipopeptides using alternative material like waste. Moreover, since the separation and purification of these natural surfactants is challenging, a comprehensive understanding of their structure–activity relationships is still in its infancy and warrants further research.

Although previous studies have proven the low and non-toxicity of some biosurfactants, more research is needed to study the toxicity of biosurfactants prior to their use in food and pharmaceutical products. Furthermore, even though the bioactivities of the biosurfactants have been demonstrated in different studies, their stability during food processing and storage is not yet fully understood. So, further research is needed to consider the effects of different environmental factors (temperature, time, pH, oxidation, etc.) on the structure and activities of biosurfactants. The impact of delivery systems on the functionalities of biosurfactants has been overlooked in recent studies, despite the fact that this technology is highly effective in improving the stability and bioavailability of biosurfactants while reducing their toxicity. Further research is required to explore the antimicrobial, anticancer, and anti-inflammatory effects of different types of biosurfactants.

## Figures and Tables

**Table 1:** Functional properties of biosurfactants.

Functional Properties	Biosurfactant Types	Microorganism Isolates	Used Tests	Mechanisms	Results	Ref.
Antibacterial activities	Lipopeptides (surfactin, iturin and fengycin)	*Bacillus amyloliquefaciens* C-1	The disc diffusion and the broth microdilution methods, scanning electron microscope, and fluorescence microscope analysis	Destruction of the integrity and permeability of the cell membrane and cell wall leading to cellular death	Considerable inhibitory effects against *Clostridium difficile* (MIC: 0.75 μg/mL)	[10]
	Lipopeptides (Brevilaterin B)	*Brevibacillus laterosporus* S62-9	The broth microdilution method and transmission electron microscopy	Destruction of the cytoplasmic membrane, subsequent loss of intracellular material, and eventual cell death	Significant inhibitory effects against *Listeria monocytogenes* (1 μg/mL)	[11]
	Lipopeptides (surfactin)	*Bacillus velezensis* SK	Agar well diffusion assay	NR	The antimicrobial activities of the extracted lipopeptide, specifically against *B. cereus* and *Staphylococcus aureus*, were observed to be stable over a wide pH range of 2 to and thermos-stability range of 0 to 80 °C	[12]
	Rhamnolipids	NR	The micro-broth dilution method	Their amphiphilic character that permits interaction with phospholipids, modifying cytoplasmic membrane permeability due to pore formation with consequent leakage of cell components and cell death	Gram-positive bacteria are more sensitive (*S. aureus*)	[13]
	Glycolipoprotein	*Oceanobacillus* sp.	Disk diffusion method	NR	More excellent inhibition zone for *E. coli*	[14]
	Glycolipoprotein	*Lactobacillus plantarum* 60 FHE	Well diffusion agar method	NR	Greater inhibitory effects on *Staphylococcus epidermidis* and *Micrococcus luteus*	[15]
	Sophorolipid	*Candida* spp.	NR	To generate ROS to damage the microbial cell	Considerable antibacterial activities against both Gram-negative and positive pathogens (*E. coli* and *B. subtilis*)	[16]
Antifungal activities	Lipopeptide (fengycin and iturin)	*Bacillus amyloliquefaciens*	Agar disk diffusion test	To inhibit the mycelial growth and sporulation of *Colletotrichum gloeosporioides* and *Fusarium oxysporum*	*Fusarium oxysporum* exhibits higher sensitivity to fengycin compared to iturin, while fengycin demonstrates more potent inhibitory effects against *Colletotrichum gloeosporioide*	[17]
	Lipopeptides (mycosubtiliin and surfactin)	*Bacillus* sp.	Broth microdilution method	NR	Significant antifungal activities against *Paecilomyces variotti* and *Byssochlamys fulva*	[18]
Antiviral activities	Lipopeptides (fengycin)	*Bacillus amyloliquefaciens* PPL	Western blotting	NR	Antiviral activities against Cucumber mosaic virus	[19]
	Rhamnolipids	*Pseudomonas gessardii* M15	Plaque reduction assays PCR, fluorescent microscopy, and Transmission electron microscopy	To solvate and disrupt lipid-based viruses and inhibit the attachment of virus to the host cell	Antiviral activities against HSV-1 and SARS-CoV-2	[20]
	Glycolipids	*Azadirachta indica*	Plaque reduction assay and Eliza	Anti-viral effects against HSV	Inhibits inflammatory cytokines	[21]
Antibiofilm activities	Glycolipid	*Bacillus pumilus* SG	Microtiter plate method	90% *P. aeruginosa* biofilm reduction	The amphipathic properties of glycolipids can increase bacterial surface hydrophobicity, disrupt lipid packing, alter the cell membrane permeability, and finally reduce microbial adhesion to solid surfaces.	[22]
		*Lactobacillus rhamnosus*	Microtiter plate method	~71% *Bacillus subtilis* biofilm reduction	Disrupts the integrity of the cell walls and inhibits EPS production	[23]
	Lipopeptides (bacillomycin D)	*Bacillus subtilis* TR47II	Microtiter plate method	~90% *C. albicans* biofilm reduction	Damages biofilm in a concentration-dependent manner	[24]
	Glycolipoprotein	Acinetobacter M6	Microtiter plate method	~83% MRSA biofilm destruction	NR	[25]
	Lipopeptides	*Halomonas venusta* PHKT	NR	More than 80% biofilm eradication against *E. coli, Staphylococcus aureus*, and *Candida albicans*	NR	[26]
Antioxidant activities	Lipopeptides	*Bacillus methylotrophicus* DCS1	DPPH	Radical scavenging activities	NR	[27]
		*Bacillus subtilis* SPB1	β-Carotene bleaching assay, DPPH, FRAP, and Ferrous ion chelating assay	Neutralizes free radicals	Enables the reaction of hydrocarbons in the hydrophobic chain with the free radicals	
		*Bacillus subtilis* #309	DPPH, ABTS, and FRAP	Free radical scavenging capacity	This ability is dependent on the molecular structure of surfactin, specifically regarding the presence of hydroxyl groups, hydrophobic amino acids, and acidic amino acids	[28]
		*Bacillus subtilis* VSG4 and *Bacillus licheniformis* VS16	DPPH and hydroxyl radical scavenging assays	Free radical scavenging capacity	NR	[29]
	Glycolipid	*Saccharomyces cerevisiae* URM 6670	DPPH, TAC capacity, Sequestration of Superoxide Ion	Superoxide ion inhibition	The biosurfactant is a potential antioxidant at concentrations above 5000 μg/mL	[30]
Anti-cancer activities	Lipopeptides (surfactin)	*Bacillus subtilis* 573	MTS	Significant anti-breast cancer potential	Inhibits cell proliferation (T47D and MDA-MB-231 cell lines)	[31]
	Glycolipoprotein	Acinetobacter M6	NR	Significant anti-lung cancer potential	Reduces cell viability and induces cell cycle arrest at the G1 phase	[25]
	Rhamnolipids	*Pseudomonas aeruginosa* RA5	Sulforhodamine B test	Considerable anticancer activities against breast and leukemia cancer	NR	[32]
Anti-inflammatory activities	glycolipid	*Rhodococcus ruber* IEGM 231	Western Blot Analysis	Significant reduction of pro-inflammatory cytokines	Inhibits the production of IL-12, IL-18, and ROS	[33]
	Sophorolipids	*Candida bombicola*	Western Blot Analysis	Significant reduction of pro-inflammatory cytokines	Reduces the IgE level, mRNA expression of TLR-2, IL-6, and STAT3	[34]
	Lipopeptides	*Staphylococcus aureus*	Western blot analysis and Eliza	Significant reduction of pro-inflammatory cytokines	Increases STAT-3 phosphorylation and the expression of heme oxygenase-1 (HO-1)	[35]

Abbreviations: EPS: extracellular polymeric substances; DPPH: 2,2-diphenyl-1-picrylhydrazyl; ABTS: 2,2-azinobis (3-ethyl-benzothiazoline-6-sulfonic acid; FRAP: ferric reducing antioxidant power; IL-12: interleukin-12; IL-18: interleukin-18; ROS: reactive oxygen species; HO-1: heme oxygenase-1; IgE: immunoglobulin E; HSV: herpes simplex virus; MRSA: methicillin-resistant *S. aureus*; MTS: (3-(4,5-dimethylthiazol-2-yl)-5-(3-carboxymethoxyphenyl)-2-(4-sulfophenyl)-2H-tetrazolium) method; NR: not reported.

**Table 2:** The advantages and drawbacks of screening methods to detect biosurfactant—producing microorganisms.

Biosurfactant Producing Screening	Advantages	Disadvantages
Oil-spreading assay	Speed Simplicity No required special tools Reliability Minimum samples required	Non-quantitative Time-consuming
Microplate assay	Speed Simplicity Accuracy Minimum samples required	Non-quantitative Time-consuming
Du-Nouy-Ring method	Simplicity Accuracy Direct measurement	Requires special tools Non-simultaneous measurement Significant samples required
Drop collapse assay	Speed Simplicity Reproducibility Qualitative method Minimum samples required	Non-quantitative Difficult observation
Emulsifying capacity assay (E24)	Simplicity Accuracy	Requires significant sample size Time-consuming Semi-quantitative
CTAB Agar Plate	Semi-quantitative	Difficult observation Restricted biosurfactant detection (e.g., anionic surfactants and glycolipids)
Homolysis	Simplicity	Inaccuracy Restricted biosurfactant detection Poor specificity
